# Editorial: Epigenetic Regulation of Stem Cell Plasticity in Tissue Regeneration and Disease

**DOI:** 10.3389/fcell.2020.00082

**Published:** 2020-02-19

**Authors:** Fulvio Chiacchiera, Lluis Morey, Chiara Mozzetta

**Affiliations:** ^1^Department of Cellular, Computational and Integrative Biology (CIBIO), University of Trento, Trento, Italy; ^2^Sylvester Comprehensive Cancer Center, Miami, FL, United States; ^3^Department of Human Genetics, University of Miami Miller School of Medicine, Miami, FL, United States; ^4^CNR -Institute of Molecular Biology and Pathology, c/o Department of Biology and Biotechnology “C. Darwin”, Sapienza University, Rome, Italy

**Keywords:** stem cells, epigenetics, regeneration, cancer epigenetics, iPSC (induced pluripotent stem cell)

Several adult tissues are endowed with remarkable regenerative capacities. Their architecture and functionality are preserved through the presence of a dedicated stem cell pool that fuels tissue regeneration during homeostasis or after acute injuries. Therefore, preserving tissue-specific cell identity by regulating proliferation and differentiation is key to maintain the correct function of the tissue. All the external stimuli that control tissue homeostasis and repair converge on chromatin to activate or repress specific set of genes. To coordinate the different transcriptional programs that regulate such processes, specific multiprotein complexes with specific enzymatic activities play a pivotal role. All these epigenetic activities shape the proper chromatin landscape allowing stem cell plasticity both *in vivo* and *in vitro* (Avgustinova and Benitah, 2016). The key role of the epigenetic mechanisms to preserve stem-cell identity is emphasized by their involvement in several diseases and during development. Indeed, mutations affecting the activities of chromatin modifying complexes are frequently incompatible with life or result in severe developmental defects. Several oncogenic mutations affecting chromatin modifying molecules have been also identified for being responsible for cancer initiation, progression, and invasion. Cancer cells are addicted to mutations in genes encoding epigenetic factors or to their aberrant activity when mutated (Cavalli and Heard, 2019). During the last decade, small compounds designed to target epigenetic factors to either modulate or block their activity, paved the way for the development of novel therapeutic interventions and for improved cell-based therapy (Dawson, 2017). In this Research Topic some of these aspects are presented and discussed considering the most recently published evidences.

A milestone of the last 20 years of scientific accomplishments is the possibility to modulate cell fate by reprogramming the transcriptional and epigenetic landscape of adult stem and differentiated cells. The potential to generate specific cell types capable of replacing damaged or aged human tissues is the coveted vision of regenerative medicine. Human induced pluripotent stem cells (iPSC) hold promise to fulfill this vision, as they display the potential to differentiate into any specific cellular lineage (Rowe and Daley, 2019). Clinically, this breakthrough finding is one of the most promising discoveries in regenerative medicine, opening up unprecedented opportunities for patient- specific stem cell-based therapies such as tissue replacement and drug discovery. Such a powerful approach however does not come without risks. The reprogramming process *per se* and the remarkable transcriptional and epigenetic changes that are induced, exposes cells to mutational events and accumulation of epigenetic abnormalities. This imposes a careful evaluation that a stable genetic and (epi)genomic state is reacquired. Perrera and Martello focus their attention on genomic imprinting. They thoroughly review the epigenetic alterations that could affect imprinted loci, discussing their impact on the reprogramming process, and presenting the potential hazards for clinical applications.

An extensive rewiring of the epigenetic landscape is observed during cancer formation and progression. Oncogenic mutations are frequently detected in chromatin modifying complexes, including SWI/SNF, Polycomb and COMPASS/COMPASS-Like complexes (Feinberg et al., 2016). Chan and Chen discuss the extensive rearrangements imposed to the epigenetic landscape by oncogenic MLL-fusion proteins causing leukemia. They review the roles of MLL-containing complexes during homeostasis and tumor formation and provide a compelling overview of the pharmacological strategies currently under investigation that exploit the specific vulnerabilities associated with MLL-fusion proteins-induced leukemia.

Histone- and DNA-demethylating enzymes belonging respectively to the family of the Jumonji-C domain-containing histone demethylases (JHDMs) and the ten-eleven translocation (TET) proteins represent another layer of epigenetic control and provide additional opportunities to shape chromatin landscape. While JHDMs catalyse the removal of mono-, di-, and tri- methylation marks on the lysine residues of multiple histones, TET enzymes catalyse DNA demethylation through the successive oxidation of 5-methylcytosine (5mC) to 5-hydroxymethylcytosine (5hmC), 5-formylcytosine (5fC), and 5-carboxylcytosine (5caC) (Schubeler, 2015). Being Fe^2+^- and α-ketoglutarate-dependent dioxygenases, the activity of both these class of enzymes can be enhanced by simply using Vitamin C. Chong et al. discuss how different applications of vitamin C boost JHDMs and TETs activity. From somatic cell reprograming to the regulation of cancer cell epigenome the authors extensively reviewed Vitamin C activity, discussing new perspectives of how nutrients and metabolism can modulate chromatin state.

The role of metabolic pathways in modulating the epigenetic landscape of the cells is also the topic of the review from Purohit and Dhawan. The authors comprehensively reviewed the recent findings on the metabolic pathways sustaining muscle stem cells providing an overview of the interdependency between epigenetic modifications and metabolic reactions in preserving adult skeletal muscle homeostasis.

Altogether the reviews collected in this Research Topic provide an overview of some important epigenetic mechanisms involved in stem cell maintenance, highlighting new potential opportunities for the development of novel approaches in cancer treatments, degenerative diseases, and stem cell-based applications. Pharmacological manipulation of the epigenetic landscape is now one of the most attractive and intense field of investigation. Several compounds, the so-called “epi-drugs,” are currently tested in clinical trials as potential treatments in different cancer types. Of note, a number of previously developed compounds have been shown effective in reverting aberrant disease-associated epigenetic states, indicating that drug repurposing might likely accelerate their transition toward clinical application.

## Author Contributions

All authors listed have made a substantial, direct and intellectual contribution to the work, and approved it for publication.

### Conflict of Interest

The authors declare that the research was conducted in the absence of any commercial or financial relationships that could be construed as a potential conflict of interest.
